# Circulating plasma protein biomarkers associated with risk of negative clinical outcomes in virally suppressed people with HIV receiving medications for opioid use disorder

**DOI:** 10.3389/fmolb.2026.1728953

**Published:** 2026-04-28

**Authors:** Livio Azzoni, Liao Zhang, Kaiyi Zhu, Matthew Fair, Emily Hiserodt, Karam Mounzer, Jeffrey J. Wallin, Luis J. Montaner, Yanhui Cai

**Affiliations:** 1 HIV Cure and Viral Diseases Center, The Wistar Institute, Philadelphia, PA, United States; 2 Gilead Sciences, Inc., Foster City, CA, United States; 3 Philadelphia FIGHT, Philadelphia, PA, United States

**Keywords:** dementia risk, kidney disease, medications for opioid use disorder, people with HIV, proteomics biomarkers

## Abstract

**Background:**

Opioid use impairs immune response, but the mechanism of action underlying this effect remains unknown. In people with HIV-1 (PWH), including those on suppressive antiretroviral therapy (ART), opioid use may exacerbate chronic inflammation. To address this knowledge gap, we sought to identify immune biomarkers in virally suppressed PWH on medications for opioid use disorder (MOUD), including methadone (MET) and suboxone (SUB), and to assess possible associations with clinical outcomes.

**Methods:**

The study enrolled PWH receiving ART from the Philadelphia FIGHT Treatment Center in three groups based on their medication regimen: control (no known opioid use, no MOUD, n = 14), SUB (n = 15), and MET (n = 14). Plasma samples were analyzed with the SomaScan platform for differentially expressed proteins. Based on protein signature modeling, SomaSignal tests were evaluated for clinically relevant information about participant health and risk status. Between-group differences were tested using Wilcoxon rank-sum test and beta regression; p values were adjusted for false discovery rate.

**Results:**

The mean age of participants was 48.6 years and the mean ART duration was 10.4 years, showing consistency across all groups. However, the proportion of female participants was lower in the control group (14.3%) versus the SUB (40.0%) and MET (42.9%) groups. The SomaScan assay revealed 12 unique proteins, identified by 13 SOMAmers, that were differentially expressed between the control and MOUD groups. Specifically, PWH on MOUD exhibited higher levels of HSP70, KERA, NTR1, FLRT2, sCD14, SDF-1, IGLL1, AT1B2, and ROR1, while the control group had higher levels of MMAC, IGFALS, and ELA2A (p < 0.001). ELISA analysis corroborated the findings, showing that results for HSP70, IGLL1, and sCD14 were concordant with SomaScan results. SomaSignal tests indicated that PWH receiving MET had possible higher risks for kidney disease, heart failure, and dementia, alongside trends suggesting reduced visceral fat and alcohol impact versus other groups. The increased risks for kidney disease and dementia were confirmed using beta regression analysis.

**Conclusion:**

These data suggest that PWH chronically exposed to MOUD have higher levels of circulating protein biomarkers that may be linked to increased risk of adverse clinical outcomes.

## 1 Introduction

Although antiretroviral therapy (ART) has dramatically improved outcomes for people with HIV-1 (PWH) by improving overall health, extending life expectancy, and greatly reducing the risk of HIV transmission, it does not reverse the long-term health risks associated with the infection (Deeks et al., 2013a; Klatt et al., 2013; Nakagawa et al., 2013). Even when viral replication is effectively suppressed by ART, HIV infection leads to chronic inflammation, which increases the risks of cardiovascular disease, chronic obstructive pulmonary disease, liver fibrosis, osteoporosis and fractures, chronic kidney disorders, non-AIDS-defining cancers, and neurocognitive disorders (Collins et al., 2021; Marcus et al., 2020; Morales et al., 2022; Nanditha et al., 2021; Paudel et al., 2024; Pourcher et al., 2020; Tsai et al., 2022; Vallée et al., 2024). The underlying mechanisms of chronic inflammation are complex, resulting from persistent low-level viral replication, microbial translocation, and dysregulation of both the innate and adaptive immune systems (Deeks et al., 2013b; Klatt et al., 2013; Nasi et al., 2017).

The interactions between opioid use and HIV infection are complex; worldwide, people who inject drugs (PWIDs) are at elevated risk of HIV infection due to multiple factors including needle sharing, sex work, and housing insecurity (Degenhardt et al., 2017; Harm Reduction International, 2023; Joint United Nations Programme on HIV/AIDS UNAIDS, 2023). Overall, PWH comprise a vulnerable population, many with a history of injection or other drug use contributing to HIV acquisition and opioid use disorder (OUD) (Cernasev et al., 2020; Schranz et al., 2018; Tsui et al., 2021). The dual burden of managing OUD with medications for opioid use disorder (MOUDs) and managing HIV with ART underscores the importance of integrated treatment approaches (Schranz et al., 2018).

Opioid use impairs immune response through interaction with µ-opioid receptors (MORs) on the surface of various immune cells, including T, B, NK, and myeloid cells, which alters their function and potentially compromises the body’s ability to fight infections and regulate inflammation (Ament et al., 2024; Azzoni et al., 2020; Garcia et al., 2012; Roy et al., 2011; Zaki et al., 2006; Zhu et al., 2016). Signaling pathways involving NF-κB and PKC ζ are triggered, leading to the secretion of proinflammatory cytokines, impaired Th1 responses, and reduced antibody production (Happel et al., 2011; Ninković and Roy, 2013; Roy et al., 2011; Saurer et al., 2004). Recent studies have demonstrated that opioid use is linked to increased levels of biomarkers of chronic inflammation and indicators of microbial translocation such as soluble CD14 (sCD14), lipopolysaccharide-binding protein, and beta-d-glucan, independent of HIV infection status (Hileman et al., 2022). Thus, chronic opioid use and/or sustained interactions with MORs may promote host mechanisms linked to immune modulation that may impact functionality and exacerbate chronic inflammation in PWH.

Currently, full (methadone, MET) and partial (buprenorphine, BUP) MOR agonists are the most prescribed MOUDs, whereas MOR antagonists like naltrexone are used less frequently (Spayde-Baker and Patek, 2023). Among MOUDs, BUP (combined with naloxone, commonly known as suboxone, SUB) in oral form or in extended-release form is often preferred due to its lower risk of overdose (Davis et al., 2025; Tingley and Mierzwinski-Urban, 2023), as well as lower licensing barriers for prescribers. While MOR agonists have proven effective in improving clinical outcomes for PWH and OUD, primarily by reducing the risk of overdose and increasing adherence to ART, their long-term use can introduce complex effects on the immune system and overall health of PWH. For instance, MET has been shown to significantly increase several inflammatory biomarkers in PWH on suppressive ART, including sCD14, IL-18 bp, TNFR-II, and IL-8 (Azzoni et al., 2022). Additionally, higher levels of the sialylated A1 glycan and a-galactosylated G0FB, both of which are linked to inflammatory states, are biomarkers altered by MET in PWH on ART (Azzoni et al., 2022). The intricate interplay of HIV, chronic inflammation, and the unknown long-term effects of MOUD use presents a unique challenge in managing the health of PWH with OUD. Furthermore, although opioid use in PWH may exacerbate chronic inflammation and possibly limit ART-mediated immune reconstitution, the mechanism of action underlying this effect remains unknown.

In this article, we present results from a pilot study to explore proteomic biomarkers in PWH who are receiving ART and MOUD versus ART only. Our goals were to identify immune biomarkers and investigate any potential links between MOUD use in PWH on ART and the risk for specific clinical outcomes, addressing a critical gap in current HIV and substance use research. This study revealed that PWH on ART receiving MOUD, particularly MET, exhibited distinct proteomic profiles with elevated levels of specific inflammatory markers including sCD14, HSP70, and IGLL1. Furthermore, our data identified biomarkers linked to an increased risk for kidney disease and dementia in this population, which may facilitate both monitoring and future therapy studies to improve clinical outcomes.

## 2 Materials and methods

### 2.1 Study design and participants

**FIGURE 1:**
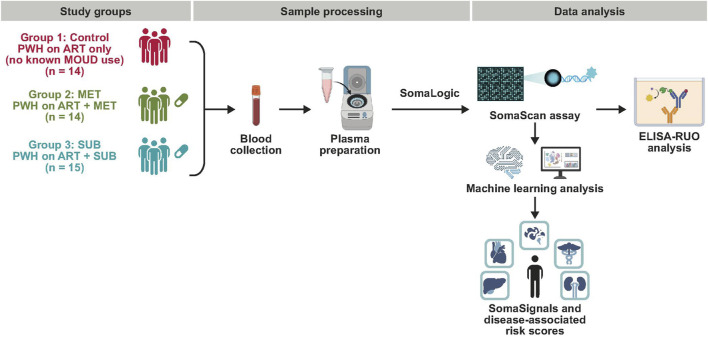
Study design and cohort overview. Schematic representation of the study workflow and participant groups. A total of 43 PWH on ART were stratified into three groups based on medication regimen: control (no known opioid use or MOUD, n = 14), MET (n = 14), and SUB (n = 15). Plasma samples were analyzed using the SomaScan v4.1 proteomic platform to identify differentially expressed circulating proteins. Selected biomarkers were further evaluated using ELISA, and predefined SomaSignal proteomic profiles were investigated to assess associations with predicted clinical outcomes. ART, antiretroviral therapy; MET, methadone; MOUD, on medications for opioid use disorder; PWH, people with HIV; RUO, research use only; SUB, suboxone/buprenorphine.

The study overview is illustrated in Figure 1. A convenience cohort of 43 PWH, all on ART, was assembled from participants recruited for multiple studies between January 2018 and October 2022 at the Jonathan Lax Treatment Center/Philadelphia FIGHT in Philadelphia, PA, United States, which provides healthcare for PWH regardless of the person’s insurance status or ability to pay. The cohort was divided into three groups based on their medication regimens: Group 1 (control) consisted of PWH receiving ART and no known opioid or MOUD use; group 2 (MET) included PWH receiving ART and daily oral MET; and group 3 (SUB) comprised PWH receiving ART and either sublingual SUB (buprenorphine + naloxone) or extended-release buprenorphine. All samples were collected under institutional review board–approved protocols.

### 2.2 Sample preparation

Whole blood was obtained from each study participant through venipuncture using acid-citrate-dextrose vacutainer tubes. Plasma was fractionated by centrifugation at room temperature, then aliquoted and stored at −80 °C until analysis. Frozen plasma samples (−80 °C) were sent to SomaLogic (Boulder, CO, United States) for SomaScan assay measurements.

### 2.3 Proteomic measurement using the SomaScan platform

Proteomic profiles were assessed at SomaLogic using the SomaScan v4.1 proteomic platform, which provided measurements on 6,628 unique human circulating proteins using 7,596 slow off-rate modified aptamers (SOMAmers) and quantified protein levels in the form of relative fluorescence units. The SomaScan assay quantitatively evaluates the proteins present in a biological sample via specific peptide-binding SOMAmer-based DNA signals. SOMAmers are unique single-stranded DNA or RNA affinity reagents that recognize folded protein-specific peptide epitopes with high affinity and specificity (Gold et al., 2010; Kraemer et al., 2024).

SomaScan v4.1 analysis followed SomaLogic’s standard pipeline including signal-to-noise filtering (≥3), hybridization normalization, and SOMAmer-specific scale factors. Before statistical analysis, the SomaScan readouts were first normalized using hybridization controls to mitigate variation within the run. This was followed by median signal normalization across pooled calibrator replicates to mitigate within-run technical variation in the calibrator signal before use in scaling calculations. The set of ratios of the calibrator reference value to the median of calibrator replicates for each SOMAmer was calculated in two ways: plate scale, the median ratio, which adjusts for overall signal intensity differences between runs; and calibration scale, the recalculated set of scale factors, one for each SOMAmer, which adjusts for SOMAmer-specific assay differences between runs. Prefiltering of analytes was performed using recommended quality-control criteria from SomaLogic and a signal-to-noise ratio >3 to increase more signals after multiple testing correction (Supplementary Table S1). No log_2_ fold-change threshold was applied to maximize discovery sensitivity in this pilot study (n = 43). Significant differences in SOMAmer-based DNA signals were denoted as false discovery rate (FDR) < 0.1 and FDR < 0.05. Log_2_ fold-change values for the 12 differential biomarkers between PWH on ART receiving MOUD versus no MOUD (controls) are reported.

### 2.4 Validation of select biomarkers using ELISA

Protein biomarkers of interest identified by SomaScan were independently analyzed using a research-use-only enzyme-linked immunosorbent assay (ELISA-RUO) at the Wistar Institute (Philadelphia, PA, United States). Plasma levels of select biomarkers from the SomaScan assay were also evaluated in duplicate using this ELISA method, following the manufacturer’s instructions. Details of the ELISA-RUO kits can be found in Supplementary Table S2. Sensitivity analyses were performed to assess the potential influence of including viremic samples or restricting to virally suppressed participants (viral load [VL] ≤ 200 copies/mL) on the ELISA results.

### 2.5 Clinical outcomes selection and prediction

Clinical outcomes were assessed using 21 predefined SomaSignal biomarker profiles derived from SomaScan data. SomaSignal profiles are machine-learning–derived risk scores developed by SomaLogic using large general-population cohorts. In this study, SomaSignal tests were used to assess relative risk trends and associations rather than to establish diagnoses. SomaScan data were input into these models to generate risk probability scores (0-1), which were then compared across groups using Kruskal–Wallis and beta regression analyses. The 21 SomaSignal tests were divided into four categories. Details on the SomaSignal tests are outlined in Supplementary Table S3. The SomaSignal kidney prognosis test utilized a 10-feature logistic regression model developed from 3,205 patients with chronic kidney disease to predict 4-year progressive chronic renal insufficiency, with a cutoff of a predicted probability ≥0.35 indicating positive risk. The SomaSignal dementia risk test employed a 25-feature accelerated failure time model developed from 11,277 subjects to estimate 20-year dementia risk. Both models were developed and validated in general populations. The SomaSignal kidney prognosis test was performed according to manufacturer’s protocol (https://somalogic.com/somasignal-tests-for-research-use/); full details are provided in the Supplementary Material.

### 2.6 Statistical analysis

Statistical analyses were conducted using R version 4.2 software. For SomaScan assay, Wilcoxon rank-sum test was used to compare differences between two groups (MOUD vs. controls). SomaScan results were independently analyzed using an ELISA-RUO assay, and differences between groups were analyzed by Wilcoxon rank-sum test and Spearman’s rank correlation test. Associations between possible clinical outcomes and protein biomarkers were determined by Wilcoxon rank-sum test, or Kruskal–Wallis test followed by Dunn’s *post hoc* test.

For SomaSignal tests, group comparisons used both Kruskal–Wallis with Dunn’s *post hoc* test and beta regression for SomaSignal probabilities. Concordance across methods was prioritized for interpretation. *P* values were adjusted for false discovery rate.

## 3 Results

### 3.1 Participant characteristics

A total of 43 participants were included in the plasma proteomics analysis, with each also undergoing 21 SomaSignal predefined biomarker profile tests for clinical outcomes. The demographic and clinical characteristics of the study participants are detailed in Table 1. The median (range) age of participants was 50.0 (25–62) years. The median (range) duration of ART was 10.0 (0.750–29.0) years (Table 1). The percentage of females was lower in the control group (14.3%) versus the SUB (40.0%) and MET groups (42.9%). Of the 43 participants, 21 (48.8%) were identified as Caucasian/White, 18 (41.9%) as African American/Black, 2 (4.7%) as Other, and 2 (4.7%) had race unknown or not reported (Table 1). CD4^+^ cell counts ranged from 132 to 1,629 cells/μL, with 39 out of 43 (90.7%) participants having undetectable VL of <50 copies/mL. Participants in the MET and SUB groups had been on MOUD for a minimum of 6 months.

**TABLE 1:** Baseline characteristics of study participants.

Characteristic	Control (ART only) (n = 14)	MET + ART (n = 14)	SUB + ART (n = 15)	Overall (N = 43)
Gender, n (%)				
Female	2 (14.3)	6 (42.9)	6 (40.0)	14 (32.6)
Male	12 (85.7)	8 (57.1)	9 (60.0)	29 (67.4)
Age, years				
Mean (SD)	46.1 (9.4)	49.2 (11.70)	50.4 (6.33)	48.6 (9.30)
Median (min, max)	47.5 (26.0, 60.0)	51.0 (25.0, 62.0)	52.0 (41.0, 61.0)	50.0 (25.0, 62.0)
Race, n (%)				
White	4 (28.6)	8 (57.1)	9 (60.0)	21 (48.8)
African American/Black	8 (57.1)	5 (35.7)	5 (33.3)	18 (41.9)
Other	2 (14.3)	0 (0)	0 (0)	2 (4.65)
Unknown	0 (0)	1 (7.14)	1 (6.66)	2 (4.65)
Years on ART				
Mean (SD)	10.2 (5.6)	12.0 (9.2)	9.0 (3.02)	10.4 (6.37)
Median (min, max)	10.0 (2.0, 19.0)	10.0 (0.8, 29.0)	10.0 (2.0, 14.0)	10.0 (0.8, 29.0)
Viral load				
<50 copies/mL, n (%)	13 (92.9)	13 (92.9)	13 (86.7)	39 (90.7)
≥50 copies/mL, n (%)	1 (7.1)	1 (7.1)	2 (13.3)	4 (9.3)
>100 copies/mL, n (%)	1 (7.1)	1 (7.1)	1 (6.7)	3 (6.9)
Max (copies/mL)	105	50,800	788	50,800

ART, antiretroviral therapy; MET, methadone; SUB, suboxone.

### 3.2 Identification of overlapping and unique proteomes in PWH receiving MOUD versus no MOUD

**FIGURE 2:**
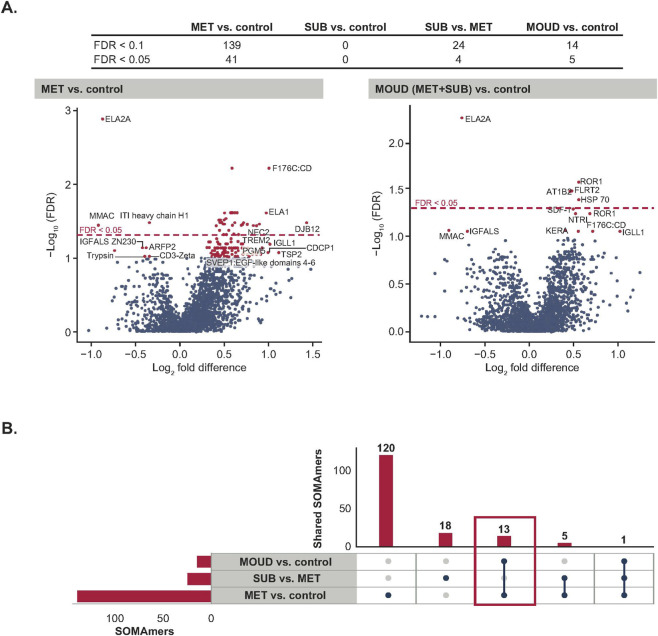
Differential plasma proteomic signals among MOUD recipients and controls. **(A)** Volcano plot showing differential SOMAmer-based DNA signals (indicating protein profiles) identified among groups (left: MET vs. control; right: MOUD vs. control). The x-axis represents log_2_ fold difference, and the y-axis represents −log_10_ adjusted p values. Statistically significant proteins were defined using FDR < 0.1 and FDR <0.05, respectively. Red dots indicate significant (FDR <0.1) differences between groups. Blue dots indicate no significant difference (FDR >0.1). The horizontal red dashed line indicates the FDR threshold (FDR = 0.05); points above this line represent significant SOMAmer-based DNA signals (FDR <0.05), while points below are non-significant (FDR >0.05). There were 139 SOMAmer-based DNA signals between MET and control with FDR <0.1, but 41 with FDR <0.05. There were 14 SOMAmer-based DNA signals between MOUD and control with FDR <0.1, but five with FDR <0.05. **(B)** Bar plot summarizing overlapping and unique differentially expressed proteins identified between MOUD and control groups, accounting for multiple SOMAmers mapping to the same target protein. Group differences were assessed using Wilcoxon rank-sum tests with FDR adjustment. FDR, false discovery rate; MET, methadone; MOUD, on medications for opioid use disorder; SUB, suboxone.

A total of 139 SOMAmer-based DNA signals were identified as significantly different (false discovery rate <0.1) between the MET and control groups (Figure 2A). In contrast, no significant SOMAmer-based DNA signals were observed between the SUB and control groups (Figure 2A). It was noted that several SOMAmers were annotated to the same protein. Among PWH receiving MOUD, 13 unique SOMAmers corresponding to 12 unique proteins were found to be more prevalent than in the control group (Figure 2B). Log_2_ fold-change values ranged from −0.91 to +0.92 (Supplementary Figure S1).

### 3.3 Differential biomarkers between PWH receiving MOUD versus no MOUD

**FIGURE 3:**
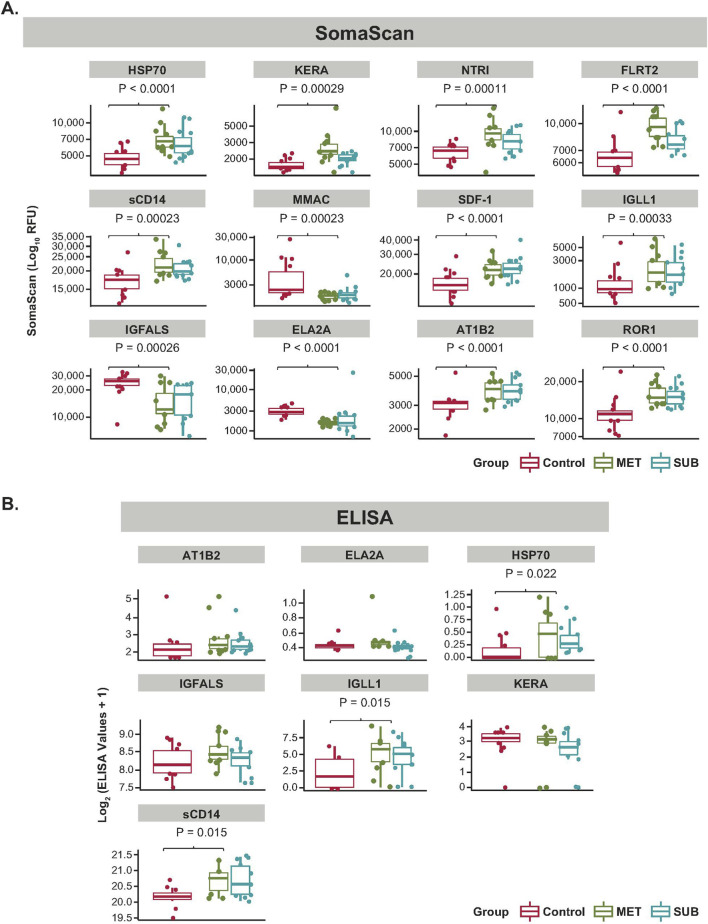
Differential biomarker levels and ELISA evaluation. **(A)** Relative levels of the 12 differentially expressed protein biomarkers identified by SomaScan in the control, MET, and SUB groups. Bars represent group median with interquartile range, and group comparisons were performed using Wilcoxon rank-sum tests with FDR adjustment (significance threshold FDR < 0.1). **(B)** Enzyme-linked immunosorbent assay (ELISA-RUO) measurements for seven selected biomarkers (HSP70, IGLL1, sCD14, KERA, IGFALS, ELA2A, and AT1B2) in the three groups. ELISA confirmed significant differences between MOUD-exposed and control participants for HSP70, IGLL1, and sCD14 (adjusted p = 0.022, 0.015, and 0.015, respectively, Wilcoxon rank-sum test), while other biomarkers showed non-significant trends and are interpreted as exploratory. A coverage threshold of over 70% specimens that were above detectable levels was established for the selected biomarkers. MET, methadone; MOUD, medications for opioid use disorder; PWH, people with HIV; RFU, relative fluorescence units; SUB, suboxone.

Of the 12 unique protein biomarkers, PWH receiving MOUD had higher expression levels of HSP70 (Heat shock protein family A member 1A), KERA (Keratan sulfate proteoglycan keratocan), NTR1 (Neurotensin receptor 1), FLRT2 (Fibronectin leucine-rich transmembrane protein 2), sCD14 (Monocyte differentiation antigen CD14), SDF-1 (C-X-C motif chemokine ligand 12), IGLL1 (Immunoglobulin lambda-like polypeptide 1), AT1B2 (ATPase Na+/K+ transporting subunit beta 2), and ROR1 (Receptor tyrosine kinase–like orphan receptor 1), while the control group had higher levels of MMAC (Phosphatase and tensin homolog MMAC1), IGFALS (Insulin-like growth factor binding protein acid labile subunit), and ELA2A (Chymotrypsin-like elastase 2A) (p < 0.001) (Figure 3A). Notably, IGLL1 showed the largest positive fold difference (log_2_ = 0.92) between PWH receiving MOUD compared with controls, while MMAC had the most pronounced decrease (log_2_ = −0.91) in PWH receiving MOUD compared with controls (Supplementary Figure S1). Qualitative functional annotation of 12 proteins (Supplementary Table S4) suggests immune activation, stress response, and cell adhesion pathways. Formal GO/KEGG analysis was underpowered (n = 12 proteins).

The trends identified by SomaScan analysis were independently tested and analyzed using ELISA-RUO for seven biomarkers (AT1B2, ELA2A, HSP70, IGFALS, IGLL1, KERA, and sCD14). The difference in ELISA-RUO measurements for HSP70, IGLL1, and sCD14 between PWH receiving MOUD and controls exhibited consistent directional and statistical agreement with the SomaScan analysis, with adjusted p values of 0.022, 0.015, and 0.015, respectively (Figure 3B). No significant differences were found for the other four exploratory biomarkers identified by SomaScan analysis (AT1B2, ELA2A, KERA, IGFALS). HSP70, IGLL1, and sCD14 showed cross-platform concordance (SomaScan + ELISA), supporting specificity despite potential aptamer off-target concerns for other proteins (Figure 3B). However, no correlation was detected for values obtained between ELISA-RUO and SomaScan (Supplementary Figure S2). Sensitivity analyses excluding viremic samples or restricting to virally suppressed participants (VL ≤ 200 copies/mL) yielded results consistent with the primary findings (Supplementary Tables S5, S6), indicating that inclusion of a limited number of samples from individuals with viremia did not substantially impact biomarker associations.

### 3.4 Association of SomaSignals with clinical outcomes

SomaSignal tests revealed that PWH on ART receiving MET had biomarker scores indicative of possible increased risks for kidney disease, heart failure, and dementia, along with a tendency for reduced visceral fat and alcohol impact, in comparison with controls (Table 2). SomaSignal risk scores are exploratory in PWH and require prospective validation. Of the 21 clinical outcome biomarker profiles tested, only the SomaSignal kidney prognosis test for chronic kidney disease showed significance in the Kruskal–Wallis comparison (p = 0.04), indicating that PWH on ART receiving MOUD had higher risk. The median kidney biomarker prognosis scores linked to development of progressive chronic renal insufficiency in 4 years were 0.08 (control), 0.14 (MET), and 0.12 (SUB), which suggests lower risk in the control group (Table 2). Beta regression analysis confirmed this association, with kidney prognosis risk score significantly elevated in MET (β = 0.72, p = 0.013) compared with the control group (Table 3). Similarly, dementia risk score was higher in the MET group based on the beta regression model (β = 0.57, p = 0.03); although, this association did not reach significance in the Kruskal–Wallis comparison (p = 0.10). Kruskal–Wallis tests (rank-based, non-parametric) and beta regression (probability modeling) yielded concordant results for kidney disease (p = 0.04 Kruskal–Wallis, p = 0.013 beta) and dementia risk (p = 0.10 Kruskal–Wallis, p = 0.03 beta) in the MET group. Discordant findings between tests reflect methodological differences and limited statistical power (n = 43) and should be interpreted cautiously (Tables 2, 3). MET showed elevated kidney prognosis risk (median score 0.14 vs. 0.08 controls; Kruskal–Wallis p = 0.04, beta regression p = 0.013) and dementia risk (beta regression p = 0.03), alongside cardiovascular risk trends (Tables 2, 3). These associations persisted in sensitivity analyses excluding viremic samples. The MET group showed a trend toward lower alcohol impact probability (median predicted probability 0.3 vs. 0.47 in the control group, p = 0.265), consistent with a proteomic signature associated with reduced heavy alcohol consumption (Table 2; Supplementary Table S3).

**TABLE 2:** Summary of SomaSignal clinical outcomes (from Kruskal–Wallis test).

Category	SomaSignals test	Unit	Control	MET	SUB	p value
Continuous	Body fat percentage	%	28.95 (5.2)	30.35 (8)	31.1 (11)	0.97
	Cardiorespiratory fitness - VO_2_ max	mL/kg/min	29.25 (6.2)	27.6 (4.3)	27.4 (7.9)	0.723
	Lean body mass	kg	55.85 (7.6)	53.7 (10)	54.7 (16.2)	0.763
	Resting energy rate	Calories/day	2,160.5 (222.5)	2,334 (482.2)	2,185 (474)	0.189
	Visceral fat	g	1,001 (564)	673.5 (610.2)	607 (484.5)	0.151
Likelihood	Heart failure prognosis - HFpEF - 12 months	%	2 (1.8)	2.75 (2.8)	2 (2.2)	0.291
	Heart failure prognosis - HFpEF - 6 months		1.15 (1)	1.55 (1.7)	1.2 (1.3)	0.299
	Heart failure prognosis - HFrEF - 12 months		2.5 (2)	4.85 (3.2)	3.5 (2)	0.096
	Heart failure prognosis - HFrEF - 6 months		1.45 (1.2)	2.8 (1.8)	2 (1.1)	0.09
	Primary cardiovascular risk - 4 years		2.9 (2.2)	3.4 (5.2)	2.6 (1.8)	0.628
	Secondary cardiovascular risk - 4 years		15.6 (18.8)	30 (27.6)	22.4 (15.3)	0.39
Probability	Alcohol impact	Probability	0.47 (0.3)	0.3 (0.1)	0.38 (0.2)	0.265
	Dementia risk		0.08 (0.1)	0.15 (0.1)	0.09 (0.1)	0.103
	Glucose tolerance		0.64 (0.5)	0.74 (0.6)	0.46 (0.5)	0.185
	Kidney prognosis^a^		0.08 (0.1)	0.14 (0.1)	0.12 (0.1)	0.04
	Liver fat		0.72 (0.5)	0.65 (0.5)	0.53 (0.3)	0.505

Results show median (interquartile range) for each group. Kruskal–Wallis test followed by Dunn’s *post hoc* test was used to compare three groups.

^a^ Probability of developing progressive chronic renal insufficiency in 4 years.

HFpEF, heart failure with preserved ejection fraction; HFrEF, heart failure with reduced ejection fraction; MET, methadone; SUB, suboxone; VO_2_, volume of oxygen.

**TABLE 3:** Summary of SomaSignal scores for kidney disease prognosis and dementia risk from beta regression model.

SomaSignal Test	Group	Estimate^a^	Standard error	*z* value	p value	Significance^b^
Kidney prognosis^c^	Control	−2.21	0.23	−9.65	5.04E-22	***
	MET	0.72	0.29	2.49	0.013	*
	SUB	0.46	0.29	1.61	0.107	NS
Dementia risk	Control	−2.20	0.21	−10.59	3.31E-26	***
	MET	0.57	0.26	2.14	0.03	*
	SUB	0.32	0.27	1.20	0.23	NS

^a^ Estimates represent the likelihood of risk/prognosis. Negative values indicate lower risk, while positive values indicate higher risk relative to the reference group (control).

^b^ Significance indicators reflect whether each group’s estimate significantly differs from zero in the beta regression model.

^c^ Likely to develop progressive chronic renal insufficiency in 4 years.

MET, methadone; NS, not significant; SUB, suboxone.

***p < 0.001; *p < 0.05.

Other SomaSignal-derived outcomes, such as liver fat, glucose tolerance, and cardiovascular risk factors, did not reach statistical significance in the Kruskal–Wallis test (Table 2), despite showing differences between groups. Notably, the MET group tended to have higher values for several cardiovascular risk factors, while the SUB group often fell between the control group and MET group for many measures.

## 4 Discussion

The data obtained from the pilot study shed light on the intricate relationship between HIV infection, OUD, and the use of MOUD in PWH on ART, with a particular emphasis on inflammatory and immune biomarkers and their potential link to possible clinical outcomes. The combined impact of these factors on the proteome of PWH on ART undergoing MOUD treatment remains largely unexplored. Our results suggest that PWH on ART chronically exposed to MOUD exhibit elevated levels of circulating protein biomarkers, which may signal a heightened risk of adverse clinical outcomes in this unique population.

The SomaScan assay identified protein biomarkers that were uniquely or commonly altered in PWH on ART and MOUD compared with the control group on ART alone, potentially revealing MOUD-specific effects on the proteome. Notably, the differential expression of 12 unique proteins suggests that MOR agonists may alter biomarker profiles. These proteins span a range of biological functions, including immune activation (e.g., sCD14) (Azzoni et al., 2022; Leeansyah et al., 2013; Lederman et al., 2013; Sandler et al., 2011), cellular stress responses (e.g., HSP70) (Binder, 2014; Yang et al., 2014), cytokine signaling (e.g., SDF-1) (Haque et al., 2020; Kim et al., 2024; Muylaert et al., 2016), and neural or cell adhesion pathways (e.g., FLRT2, NTR1, AT1B2) (Boer et al., 2010; Bugni et al., 2012; Fang et al., 2023; Mauri et al., 2017; Zhou et al., 2015). MMAC (also known as PTEN) and ELA2A appear to play important regulatory roles in immune and inflammatory processes (Chen and Guo, 2017; Esteghamat et al., 2019; Motta et al., 2021; Taylor et al., 2019; Zou et al., 2011). Supplementary Table S6 provides an overview of the broad immune and inflammatory functions of these proteins. Larger proteomic studies with expanded protein sets are needed for formal pathway enrichment analysis.

Some of the detected biomarkers have been previously associated with opioid exposure, including sCD14 and HSP70, both of which have been shown to be upregulated by MOUD (Azzoni et al., 2022; Yang et al., 2014). The elevated levels of HSP70, KERA, NTR1, FLRT2, sCD14, SDF-1, IGLL1, AT1B2, and ROR1 in PWH on ART receiving MOUD could be indicative of chronic inflammation or immune dysregulation, which are known to persist in PWH even with effective viral suppression on ART (Deeks et al., 2013a). Elevated plasma levels of sCD14, which serve as a marker of myeloid activation and/or microbial translocation linked to HIV infection outcomes (Deeks et al., 2013a), have been observed with MET usage in PWH on ART (Azzoni et al., 2022; Eisenstein, 2011; Lederman et al., 2013; Leeansyah et al., 2013; Sandler et al., 2011). The elevated levels of HSP70 in PWH receiving MOUD corroborate findings from animal studies demonstrating that morphine causes an overexpression of HSP70 in mice (Yang et al., 2014), suggesting a potential common mechanism across different opioids. Similarly, the elevated SDF-1 levels align with previous research showing that SDF-1 was a predictor of lifetime pathological use of cocaine (Araos et al., 2015). Several of the differentially expressed proteins, including FLRT2 and NTR1, are involved in cell signaling and adhesion processes. FLRT2 has been shown to regulate monocyte-to-macrophage differentiation and enhance macrophage adhesion and migration (Choi et al., 2022; Fang et al., 2023), while NTR1 influences inflammatory signaling and pain pathways, and may interact with opioid receptors, potentially modulating addiction-related signaling (Bugni et al., 2012; Feng et al., 2015; Liu et al., 2016; Wu et al., 2012). Their altered expression in PWH receiving MOUD suggests potential adaptations in cell signaling and adhesion processes in response to chronic opioid exposure. Furthermore, the differential expression of AT1B2 is particularly noteworthy, as this protein may influence addiction vulnerability across different drug classes (Gritz et al., 2016).

In contrast, MMAC, IGFALS, and ELA2A levels were lower in the MOUD group as compared with the ART only control group. MMAC, a tumor suppressor protein involved in cell signaling, was found to mediate the silencing of integrated HIV-1 DNA *in vitro* (Koul et al., 2002; Zou et al., 2011). Dysregulation of PTEN may influence both innate and adaptive immune responses and may lead to abnormal cell proliferation, survival, differentiation, energy metabolism, and alteration of cellular architecture and mobility (Chen and Guo, 2017). IGFALS is crucial for the binding and bioavailability of IGF. Clinically, plasma IGF and related proteins have been shown to be dysregulated in PWH (Suh et al., 2015). Low levels of IGFALS have been described in children with HIV compared with those without HIV and may be associated with poor growth in this population (Mudibo et al., 2024). ELA2A is secreted from the pancreas as a zymogen (Kawashima et al., 1987; Thrower et al., 2006), and exocrine pancreatic insufficiency is common in PWH on ART (Price et al., 2005; Yilmaz and Hagberg, 2018).

The independent investigation of selected biomarkers using an ELISA-RUO assay confirmed the noted differences in the levels of HSP70, IGLL1, and sCD14 levels between PWH receiving MOUD and controls. The consistent directional and statistical agreement across platforms, which support their robustness, suggests that these proteins could be useful biomarkers for MOR agonist exposure in PWH.

The clinical outcomes predicted by SomaSignal were selected for analysis utilizing machine learning and data prediction algorithms, providing a novel approach to risk assessment in this population. The SomaSignal analysis revealed possible risk trends for PWH receiving MET, including higher risks for kidney disease and dementia compared with PWH not receiving MOUD. These findings align with previous studies suggesting that chronic opioid use, even in the context of MOUD, may contribute to accelerated aging and increased risk of comorbidities (Gunn et al., 2020; Kovacs et al., 2015; Yang et al., 2013). Furthermore, our analysis indicated that PWH receiving MOUD had a higher likelihood of developing progressive chronic renal insufficiency within 4 years. This observation emphasizes the necessity for more vigilant monitoring of kidney function in PWH receiving MOUD, as chronic kidney disease is already a significant concern in this susceptible population (Deeks et al., 2013a).

The elevated kidney disease and dementia risk signals observed in the MET group could plausibly reflect several mechanisms: (a) methadone-specific effects, where full mu-opioid agonism may drive immune activation (elevated sCD14, HSP70) and stress responses that overlap with kidney and cardiovascular disease pathways; (b) persistent HIV effects, as even with ART suppression, PWH exhibit chronic inflammation and endothelial dysfunction that may interact with MOUD exposure; (c) unmeasured comorbidities or confounders, including limited baseline data on hypertension, diabetes, smoking, or baseline estimated glomerular filtration rate, as well as demographic imbalances (higher proportion of females in MET/SUB groups); or (d) SomaSignal model limitations, as these models were trained on general populations and may miscalibrate risk in PWH with unique proteomic profiles. The cross-sectional design of this study precludes causal attribution, and prospective studies are essential to clarify these associations.

The lower alcohol impact score observed in the MET group (median predicted probability 0.3 vs. 0.47 in the control group) may reflect methadone-mediated reductions in alcohol use via reward pathway modulation, proteomic adaptations unrelated to actual alcohol consumption behavior, or selection bias toward participants with better overall substance use control who are stable on methadone. However, our dataset lacks direct alcohol use measures (e.g., self-report, phosphatidylethanol, gamma-glutamyl transferase) to validate the proteomic prediction, limiting mechanistic interpretation of this finding.

The biomarkers found to be linked with adverse clinical outcomes in PWH on ART receiving MOUDs also raise the concern of risk profiles after therapeutic interventions such as shifting from MET or SUB to MOR antagonists such as naltrexone.

This research presents several limitations. First, the study was constrained by a small sample size. The interpretation of the results is limited due to the absence of data for individuals actively using opioids alone or in combination (polydrug use). While stratification by age, viral load, or comorbidities would be informative, further stratification in this cohort would substantially reduce statistical power and risk overfitting. Larger cohorts are needed to identify effect modifiers. In addition, while the methods minimized the influence of outliers, it is important to consider that one individual in each group had VL > 50 copies/mL, which might affect the results. A sensitivity analysis conducted by eliminating the high viremia sample confirmed that ELISA results remained significant, maintaining the observed risk associations for kidney disease and dementia (Supplementary Table S3). Due to its exploratory nature, this research involved a cross-sectional cohort of individuals under clinical care for HIV and/or OUD, which may have led to selection bias due to the convenience sampling method employed. The cross-sectional design precludes the ability to disentangle methadone effects from HIV persistence, comorbidities, or demographic factors. Prospective cohorts with comprehensive phenotyping are needed to address these questions.

Second, while ELISA-RUO results were concordant with the results from the SomaScan assay, cross-platform comparison for the magnitude and quality of data obtained from SomaScan (relative expression units) and ELISA (pg/mL) presents challenges. SomaScan utilized aptamers to detect and identify proteins based on their unique peptidomic readouts, whereas ELISA-RUO used an antibody-binding approach to identify proteins based on their specific structural binding features.

While aptamer-based proteomics (SomaScan) may exhibit off-target binding in plasma, concordance across multiple SOMAmers per protein and independent ELISA validation for key biomarkers (HSP70, IGLL1, sCD14) support the specificity of our primary findings. The discordance observed for other biomarkers (AT1B2, ELA2A, KERA, IGFALS) between SomaScan and ELISA platforms reflects inherent differences in detection principles: aptamer-based relative quantification versus antibody-based absolute quantification, which can lead to differences in magnitude and correlation. Additionally, the research-use-only ELISA kits employed may have variable sensitivity and specificity, and the modest sample size (n = 43) limits statistical power to detect smaller effect sizes. In the proteomic discovery phase, no log_2_ fold-change threshold was applied to maximize sensitivity given the modest sample size; while this approach could reduce specificity, it was prioritized to identify candidate biomarkers for future validation. Larger studies with orthogonal validation methods are recommended to confirm these findings. Additionally, the ELISA assay used in this study is intended for research purposes only and has not been fully validated for clinical application.

Finally, the predictions made by the SomaSignal algorithm are based on data with heterogeneous populations, not limited to PWH, and those data were collected from blood with a distinct anticoagulant (EDTA) instead of citrate dextrose. The proteomic profile might be impacted given that differences in platelet count and volume have been observed between whole blood collected with EDTA and citrate anticoagulant (McShine et al., 1990).

## 5 Conclusion

In summary, this pilot study highlights how MOUD, especially MET, may affect the proteome and long-term health outcomes of PWH on ART. Independent ELISA evaluation confirmed that HSP70, IGLL1, and sCD14 showed consistent directional and statistical agreement with SomaScan findings, supporting their robustness as potential biomarkers for MOR agonist exposure in PWH. The data suggest that PWH on ART chronically exposed to MOR agonists exhibit elevated levels of circulating protein biomarkers, which may indicate a heightened risk of adverse clinical outcomes. PWH on chronic MOUD, particularly MET, exhibit proteomic signatures associated with kidney disease and dementia risk in exploratory analyses requiring prospective validation. More extensive longitudinal studies are required to validate these results and clarify the underlying mechanisms behind the observed associations.

## Data Availability

All relevant data are included in the article/Supplementary Material. The raw data supporting the conclusions of this article will be made available by the authors, without undue reservation.
