# Supplementary material for: Circulating plasma protein biomarkers associated with risk of negative clinical outcomes in virally suppressed people with HIV receiving medications for opioid use disorder
Source: Front Mol Biosci. 2026 Apr 28;13:1728953. doi: 10.3389/fmolb.2026.1728953 (PMC13160842; doi:10.3389/fmolb.2026.1728953)
Supplement: Supplementary file 1 [file Supplementaryfile1.docx]

# Circulating plasma protein biomarkers associated with risk of negative clinical outcomes in virally suppressed people with HIV receiving medications for opioid use disorder

Livio Azzoni^1^, Liao Zhang^2^, Kaiyi Zhu^2^, Matthew Fair^1^, Emily Hiserodt^3^, Karam Mounzer^3^, Jeffrey J. Wallin^2^, Luis J. Montaner^1^, and Yanhui Cai^2^

^1^HIV Cure and Viral Diseases Center, The Wistar Institute, Philadelphia, PA, USA

^2^Gilead Sciences, Inc., Foster City, CA, USA

^3^Philadelphia FIGHT, Philadelphia, PA, USA

# Supplementary Materials

# Supplementary Methods 1. Detailed protocol for the SomaSignal kidney prognosis test

The SomaSignal kidney prognosis test predicts the likelihood of progressive chronic renal insufficiency (PCRI) development within 4 years in chronic kidney disease patients. Validated using EDTA plasma from individuals with estimated glomerular filtration rate (eGFR) 10.6-86.4 mL/min/1.73 m^2^, it was developed using diverse North American samples from individuals aged 23-75 years. The test employed a 10-feature logistic regression model based on protein patterns to estimate PCRI likelihood, defined as 50% eGFR decline, dialysis initiation, eGFR <15 mL/min/1.73 m^2^, or transplant candidacy. The model was developed from a multisite observational study (1) of 3205 US participants (ages 22-76 years, chronic kidney disease stages I-V), and used a 0.35 probability cutoff. Results were reported as a probability of developing PCRI in 4 years and a predicted class (negative/positive). The SomaSignal dementia risk test predicted 20-year dementia risk. It was validated using EDTA plasma samples from individuals without known dementia at the time of blood draw, aged 49-73, from diverse racial and ethnic backgrounds in North America. The test utilized an accelerated failure time (AFT) model with a Weibull distribution, incorporating 25 SOMAmer measurements. Developed from an observational study of 11,277 US participants (2), it estimated dementia risk across various types, including Alzheimer’s, cerebrovascular, Lewy body–related, and dementias of uncertain etiology. Dementia diagnosis was adjudicated through cognitive assessments, interviews, and medical records. The model output provided an absolute probability (0-1) of dementia diagnosis within 20 years.

# Supplementary Tables and Figures

## Supplementary TABLE 1. SomaLogic data quality control.

|  | **Samples**  **(43)** | **Analytes**  **(7596)** | **Signal/noise (>3)** |
| --- | --- | --- | --- |
| Flagged | 0 | 203 | 3840 |
| Passed | 43 | 7396 | 3756 |

## Supplementary TABLE 2. ELISA-RUO kit information.

| **Candidate** | **Name** | **Vendor** | **Catalog #** |
| --- | --- | --- | --- |
| HSP-70 | Heat shock protein family A (Hsp70) member 1A | Thermo Fisher Scientific | BMS2087 |
| KERA | Keratan sulfate proteoglycan keratocan | Biorbyt | orb564293 |
| NTR1 | Neurotensin receptor 1 | MyBioSource | MBS066168 |
| FLRT2 | Fibronectin leucine-rich transmembrane protein 2 | Thermo Fisher Scientific | EH195RB |
| sCD14 | Monocyte differentiation antigen CD14 | Biotechne | DC140 |
| MMAC | Phosphatase and tensin homolog MMAC1 | Abcam | ab206979 |
| SDF-1 | C-X-C motif chemokine ligand 12 | Abcam | ab100637 |
| IGLL1 | Immunoglobulin lambda-like polypeptide 1 | MyBioSource | MBS9341354 |
| IGFALS | Insulin-like growth factor binding protein acid labile subunit | MyBioSource | MBS2020386 |
| ELA2A | Chymotrypsin-like elastase 2A | MyBioSource | MBS109433 |
| AT1B2 | ATPase Na+/K+ transporting subunit beta 2 | MyBioSource | MBS9333609 |
| ROR1 | Receptor tyrosine kinase–like orphan receptor 1 | MyBioSource | MBS2000330 |

RUO, research use only.

## Supplementary TABLE 3. Explanation for SomaSignal tests.

| **SomaSignal tests** | **Unit** | **Category** | **Notes** |
| --- | --- | --- | --- |
| Body fat percentage | % | Continuous | Predicted fat percentage |
| Cardiorespiratory fitness - VO_2_ max | mL/kg/min |  | Predicted value for VO_2_ max in mL/kg/min |
| Lean body mass | kg |  | Predicted body mass |
| Resting energy rate | Calories/day |  | Provides resting energy expenditure in calories per day (cal/day) |
| Visceral fat | g |  | Grams of visceral fat |
| Heart failure prognosis - HFpEF - 12 months | % | Likelihood | Heart failure with preserved ejection fraction. Likelihood of all-cause death within 6 months and 1 year |
| Heart failure prognosis - HFpEF - 6 months |  |  |  |
| Heart failure prognosis - HFrEF - 12 months |  |  | Heart failure with reduced ejection fraction. Likelihood of all-cause death within 6 months and 1 year |
| Heart failure prognosis - HFrEF - 6 months |  |  |  |
| Primary cardiovascular risk - 4 years |  |  | A risk estimate (in percentage) of the likelihood of a primary/secondary cardiovascular event within 4 years of blood sample collection |
| Secondary cardiovascular risk - 4 years |  |  |  |
| Alcohol impact | Probability | Probability | PP of consuming 14 or more units of alcohol per week. PP≥0.5: high impact |
| Dementia risk |  |  | PP of dementia diagnosis within 20 years. PP≥0.22: high risk; PP≤ 0.07: low risk |
| Glucose tolerance |  |  | Predicts if a person has normal or impaired glucose tolerance. PP<0.5: normal |
| Kidney prognosis |  |  | PP of developing progressive chronic renal insufficiency in 4 years.  PP<0.35: negative; PP≥0.35: positive |
| Liver fat |  |  | Estimates the presence or absence of excess liver fat. PP<0.5: no excess liver fat |

HFpEF, heart failure with preserved ejection fraction; HFrEF, heart failure with reduced ejection fraction; MET, methadone; PP, predicted probability; SUB, suboxone; VO_2_, volume of oxygen.

## Supplementary TABLE 4. Literature-based functional annotation of differentially expressed proteins.

| **Biomarker** | **Role in immune/inflammatory pathways** | **Possible link to opioid use** | **Reference** |
| --- | --- | --- | --- |
| HSP70 | Facilitates antigen presentation and, when extracellular, acts as a danger signal that can stimulate immune responses and potentially contribute to inflammation | Morphine induces overexpression of HSP70 in mice | (3,4) |
| KERA | Structural component of extracellular matrix; involved in collagen organization, corneal transparency, and wound healing. Involved in cell signaling and regulation of neural development and repair. May help regulate chemokine gradients needed for neutrophil migration. Can provide both inhibitory and stimulatory cues for axon growth and guidance | No known link is documented | (5,6) |
| NTR1 (NTSR1) | Modulates inflammatory responses, pain signaling, and tumor development by influencing cytokine expression and cellular transformation processes | Forms heterodimers with kappa opioid receptor (KOR), shifting KOR signaling from G protein-dependent to β-arrestin-2-dependent pathways  KOR is known to play a role in addiction processes. The altered signaling due to NTR1 interaction could influence addictive behaviors or responses to drugs of abuse | (7-10) |
| FLRT2 | Adaptations in cell signaling and adhesion processes. Drives monocyte differentiation into macrophages and promotes macrophage adhesion, migration, and phagocytosis | FLRT2 was identified in a gene network differentially regulated between methamphetamine and heroin, suggesting potential involvement in opioid response | (11,12) |
| sCD14 | Marker for myeloid activation and/or microbial translocation  May serve as an indicator of immune activation and inflammation that could impact brain function | Elevated levels have been associated with methadone use in PWH on antiretroviral therapy | (13-16) |
| MMAC (PTEN) | Regulates both innate and adaptive immunity, key regulator of immune cell signaling and function. Controls the differentiation, activation, and homeostasis of multiple immune cell types, including T cells, B cells, and myeloid cells. PTEN primarily functions by negatively regulating the PI3K/AKT/mTOR pathway | No known link is documented | (17-19) |
| SDF-1 | Key chemokine that regulates immune cell trafficking and stem/progenitor cell migration, and homeostasis. Plays a critical role in inflammation, tissue repair, and immune response modulation | SDF-1 levels were positively correlated with symptom severity in cocaine users; potential link to substance use severity. | (20-23) |
| IGLL1 | Crucial for early B-cell development, encoding components of the pre-B-cell receptor complex. Plays a vital role in B-cell maturation and antibody production, with defects leading to B-cell lymphopenia and potential agammaglobulinemia | No known link is documented | (24) |
| IGFALS | Serves as a potential marker for inflammation; its levels decrease during inflammatory processes and illness | No known link is documented | (25) |
| ELA2A (CELA2A) | Regulates insulin secretion, degradation, and sensitivity and reducing platelet hyperactivation, which could impact inflammatory and immune processes in the body  Increased ELA2A activity leads to upregulation of pro-inflammatory cytokines like CXCL8 and downregulation of anti-inflammatory/repair factors like TGF-β and IL-10  ELA2A activity influences the production of both pro-inflammatory and anti-inflammatory mediators | No known link is documented | (26,27) |
| AT1B2 (AMOG) | Mediates ion homeostasis across cell membranes, functions as a cell adhesion molecule, and plays a role in neuronal migration during CNS development | No known link is documented | (28,29) |
| ROR1 | Plays a critical role in regulating satellite cell proliferation during skeletal muscle regeneration, acting as a mediator between inflammatory signals and stem cell expansion in response to injury | No known link is documented | (30) |

ART, antiretroviral therapy; MOUD, medications for opioid use disorder; PWH, people with HIV-1.

## Supplementary TABLE 5. Sensitivity analysis for ELISA tests with and without high viremic sample.

|  | **With high viremic sample** | | | **Without high viremic sample** | | |
| --- | --- | --- | --- | --- | --- | --- |
| **Test** | MET vs. Control | SUB vs. Control | SUB vs. MET | MET vs. Control | SUB vs. Control | SUB vs. MET |
| AT1B2 (ng/mL) | 0.1797 | 0.4375 | 1 | 0.3038 | 0.3975 | 1 |
| ELA2A (ng/mL) | 0.0774 | 0.8956 | 0.0027 | 0.1237 | 0.8618 | 0.0052 |
| FLRT2 (ng/mL) | 1 | 1 | 1 | 1 | 1 | 1 |
| HSP70 (ng/mL) | 0.1033 | 0.0493 | 1 | 0.2033 | 0.0419 | 1 |
| IGFALS (ng/mL) | 0.1591 | 1 | 0.1957 | 0.1258 | 1 | 0.1432 |
| IGLL1 (ng/mL) | 0.0188 | 0.0794 | 1 | 0.0387 | 0.0692 | 1 |
| KERA (ng/mL) | 1 | 0.1137 | 0.371 | 1 | 0.1281 | 0.3895 |
| NTRI (pg/mL) | 1 | 1 | 1 | 1 | 1 | 1 |
| MMAC (pg/mL) | 1 | 1 | 1 | 1 | 1 | 1 |
| ROR1 (ng/mL) | 0.3163 | 1 | 1 | 0.552 | 1 | 1 |
| SDF-1 (pg/mL) | 1 | 0.3104 | 0.7277 | 1 | 0.3319 | 0.8793 |

P-values from ANOVA Dunn post-run test for joint ranking, comparing each MOUD group (MET = methadone group, SUB = suboxone group) with the control group (ART only). The analysis was performed both with high viremic samples included (left columns) and excluded (right columns). Shaded cells indicate p values that are significant (<0.5).

## **Supplementary TABLE 6. Sensitivity analysis for ELISA tests using ANOVA: all samples versus virally suppressed samples with viral load ≤200 copies/mL**.

|  | **All samples** | **Samples with viral load ≤200 copies/mL** |
| --- | --- | --- |
| **Test** | Probability > chi squared | Probability > chi squared |
| AT1B2 (ng/mL) | 0.1394 | 0.2081 |
| ELA2A (ng/mL) | 0.0031 | 0.0088 |
| FLRT2 (ng/mL) | 0.7683 | 0.7882 |
| HSP70 (ng/mL) | 0.0313 | 0.0392 |
| IGFALS (ng/mL) | 0.09 | 0.0526 |
| IGLL1 (ng/mL) | 0.0143 | 0.0215 |
| KERA (ng/mL) | 0.0926 | 0.0588* |
| NTRI (pg/mL) | 1 | 1 |
| MMAC (pg/mL) | 0.5773 | 0.5843 |
| ROR1 (ng/mL) | 0.261 | 0.4183 |
| SDF-1 (pg/mL) | 0.2426 | 0.3494 |

Values represent p values for ANOVA.
*For KERA, when analyzing only samples with viral load ≤200 copies/mL, the comparison between the suboxone (SUB) group and the control group showed significance (p<0.05). Control refers to PWH on ART with no known opioid use or MOUD.

## Supplementary FIGURE 1. Summary of fold difference for the 12 differential biomarkers between PWH on ART receiving MOUD versus no MOUD (the control group).

There were 12 unique proteins (tagged by 13 SOMAmers) that were differentially expressed between PWH receiving MOUD and the control group. Proteins elevated in MOUD include HSP70, KERA, NTR1, FLRT2, sCD14, SDF-1, IGLL1, AT1B2, and ROR1, whereas MMAC, IGFALS, and ELA2A were higher in the control group (false discovery rate <0.01). ART, antiretroviral therapy; MOUD, medications for opioid use disorder; PWH, people with HIV-1.


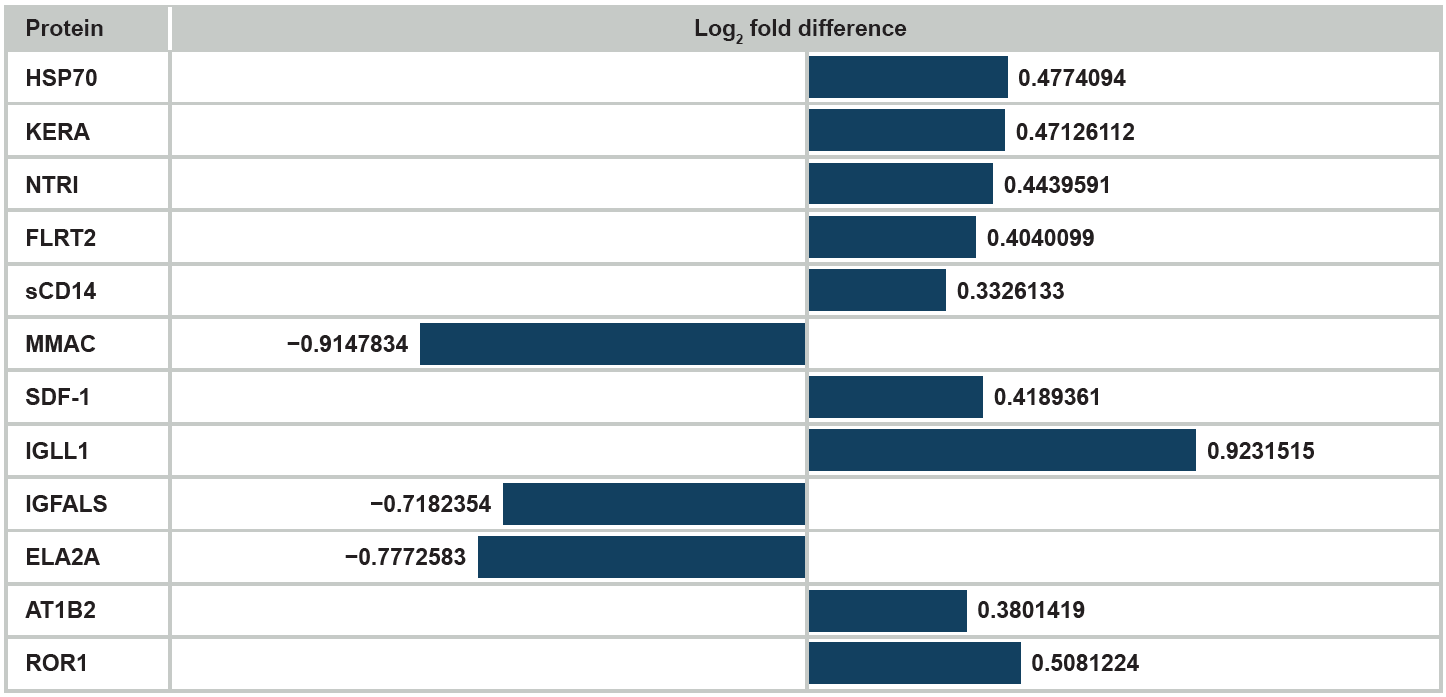


## Supplementary FIGURE 2. Correlation analysis of data from ELISA-RUO and SomaScan assay.

Each dot represents one participant’s biomarker measurement by both methods. X-axis shows log-transformed SomaScan values; Y-axis shows ELISA-RUO values in their respective units. The table displays Spearman's correlation coefficients (rho) and p values. Statistically significant correlations were found for IGLL1 (p=0.025) and sCD14 (p=0.027).
RUO, research use only.


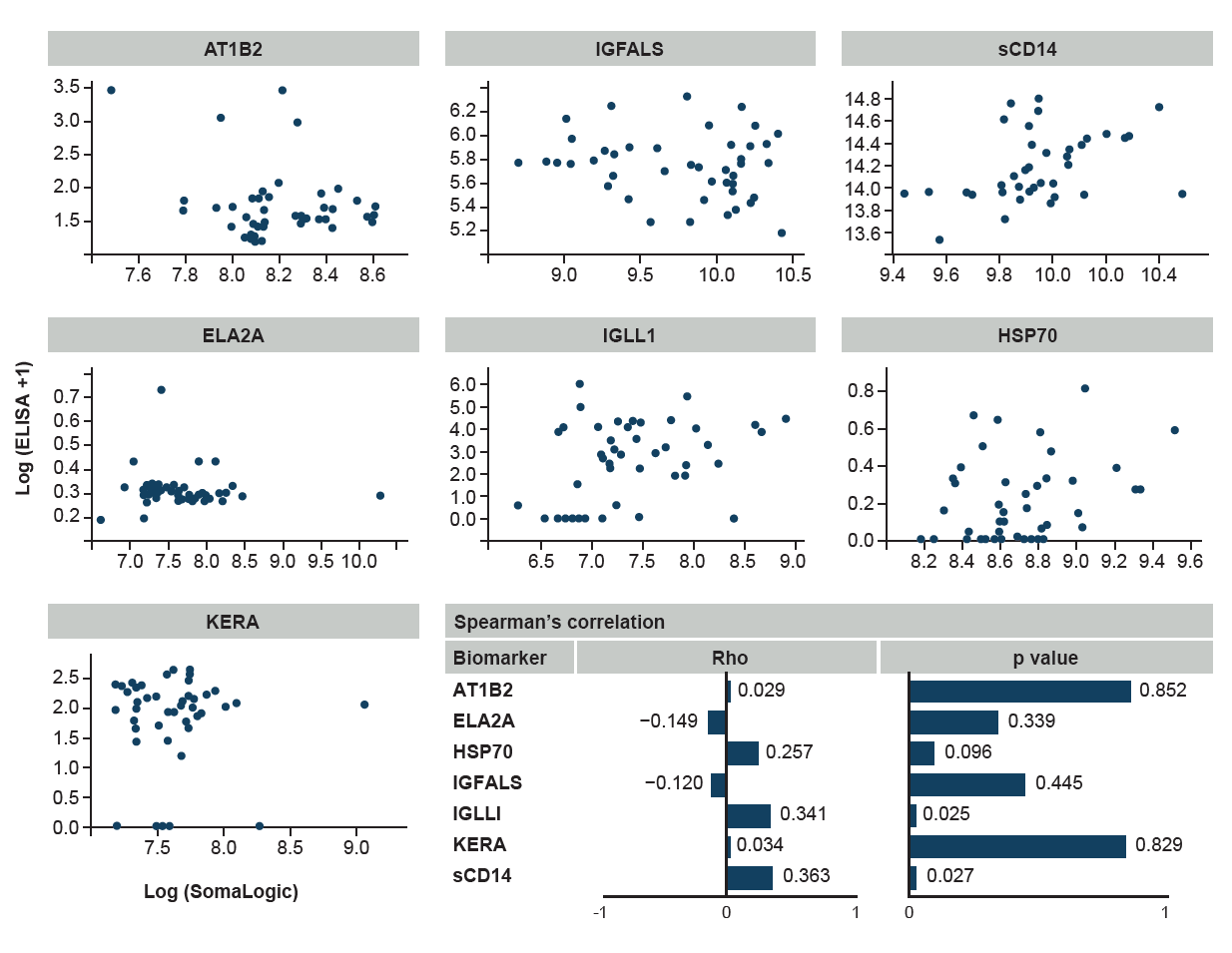


# References

1. National Institute of Diabetes and Digestive and Kidney Diseases. Diabetes Prevention Program (DPP). NIDDK Central Repository. Chronic Renal Insufficiency Cohort Study (CRIC). Available online at: <https://repository.niddk.nih.gov/study/15> (Accessed February 10, 2026).

2. Duggan MR, Paterson C, Lu Y, Biegel H, Dark HE, Cordon J, et al. The Dementia SomaSignal Test (dSST): a plasma proteomic predictor of 20-year dementia risk. *Alzheimers Dement*. (2025) 21:e14549. doi: 10.1002/alz.14549

3. Binder RJ. Functions of heat shock proteins in pathways of the innate and adaptive immune system. *J Immunol*. (2014) 193:5765-71. doi: 10.4049/jimmunol.1401417

4. Yang HY, Pu XP, Liu Y. Chronic morphine treatment induces over-expression of HSP70 in mice striatum related with abnormal ubiquitin-proteasome degradation. *Drug Alcohol Depend*. (2014) 139:53-9. doi: 10.1016/j.drugalcdep.2014.03.005

5. Caterson B, Melrose J. Keratan sulfate, a complex glycosaminoglycan with unique functional capability. *Glycobiology*. (2018) 28:182-206. doi: 10.1093/glycob/cwy003

6. Carlson EC, Lin M, Liu CY, Kao WW, Perez VL, Pearlman E. Keratocan and lumican regulate neutrophil infiltration and corneal clarity in lipopolysaccharide-induced keratitis by direct interaction with CXCL1. *J Biol Chem*. (2007) 282:35502-9. doi: 10.1074/jbc.M705823200

7. Bugni JM, Rabadi LA, Jubbal K, Karagiannides I, Lawson G, Pothoulakis C. The neurotensin receptor-1 promotes tumor development in a sporadic but not an inflammation-associated mouse model of colon cancer. *Int J Cancer*. (2012) 130:1798-805. doi: 10.1002/ijc.26208

8. Wu Z, Martinez-Fong D, Trédaniel J, Forgez P. Neurotensin and its high affinity receptor 1 as a potential pharmacological target in cancer therapy. *Front Endocrinol (Lausanne)*. (2012) 3:184. doi: 10.3389/fendo.2012.00184

9. Liu H, Tian Y, Ji B, Lu H, Xin Q, Jiang Y, et al. Heterodimerization of the kappa opioid receptor and neurotensin receptor 1 contributes to a novel β-arrestin-2–biased pathway. *Biochim Biophys Acta*. (2016) 1863:2719-38. doi: 10.1016/j.bbamcr.2016.07.009

10. Feng YP, Wang J, Dong YL, Wang YY, Li YQ. The roles of neurotensin and its analogues in pain. *Curr Pharm Des*. (2015) 21:840-8. doi: 10.2174/1381612820666141027124915

11. Fang Y, Ma K, Huang YM, Dang Y, Liu Z, Xu Y, et al. Fibronectin leucine-rich transmembrane protein 2 drives monocyte differentiation into macrophages via the UNC5B-Akt/mTOR axis. *Front Immunol*. (2023) 14:1162004. doi: 10.3389/fimmu.2023.1162004

12. Choi MR, Jin YB, Kim HN, Lee H, Chai YG, Lee SR, et al. Differential gene expression in the hippocampi of nonhuman primates chronically exposed to methamphetamine, cocaine, or heroin. *Psychiatry Investig*. (2022) 19:538-50. doi: 10.30773/pi.2022.0004

13. Azzoni L, Giron LB, Vadrevu S, Zhao L, Lalley-Chareczko L, Hiserodt E, et al. Methadone use is associated with increased levels of sCD14, immune activation, and inflammation during suppressed HIV infection. *J Leukoc Biol*. (2022) 112:733-44. doi: 10.1002/jlb.4a1221-678rr

14. Leeansyah E, Malone DF, Anthony DD, Sandberg JK. Soluble biomarkers of HIV transmission, disease progression and comorbidities. *Curr Opin HIV AIDS*. (2013) 8:117-24. doi: 10.1097/COH.0b013e32835c7134

15. Sandler NG, Wand H, Roque A, Law M, Nason MC, Nixon DE, et al. Plasma levels of soluble CD14 independently predict mortality in HIV infection. *J Infect Dis*. (2011) 203:780-90. doi: 10.1093/infdis/jiq118

16. Lederman MM, Funderburg NT, Sekaly RP, Klatt NR, Hunt PW. Residual immune dysregulation syndrome in treated HIV infection. *Adv Immunol*. (2013) 119:51-83. doi: 10.1016/b978-0-12-407707-2.00002-3

17. Chen L, Guo D. The functions of tumor suppressor PTEN in innate and adaptive immunity. *Cell Mol Immunol*. (2017) 14:581-9. doi: 10.1038/cmi.2017.30

18. Taylor H, Laurence ADJ, Uhlig HH. The role of PTEN in innate and adaptive immunity. *Cold Spring Harb Perspect Med*. (2019) 9. doi: 10.1101/cshperspect.a036996

19. Zou S, El-Hage N, Podhaizer EM, Knapp PE, Hauser KF. PTEN gene silencing prevents HIV-1 gp120(IIIB)-induced degeneration of striatal neurons. *J Neurovirol*. (2011) 17:41-9. doi: 10.1007/s13365-010-0016-z

20. Kim SY, Son MK, Park JH, Na HS, Chung J. The anti-inflammatory effect of SDF-1 derived peptide on *Porphyromonas gingivalis* infection via regulation of NLRP3 and AIM2 inflammasome. *Pathogens*. (2024) 13:474. doi: 10.3390/pathogens13060474

21. Haque N, Fareez IM, Fong LF, Mandal C, Abu Kasim NH, Kacharaju KR, et al. Role of the CXCR4-SDF1-HMGB1 pathway in the directional migration of cells and regeneration of affected organs. *World J Stem Cells*. (2020) 12:938-51. doi: 10.4252/wjsc.v12.i9.938

22. Araos P, Pedraz M, Serrano A, Lucena M, Barrios V, García-Marchena N, et al. Plasma profile of pro-inflammatory cytokines and chemokines in cocaine users under outpatient treatment: influence of cocaine symptom severity and psychiatric co-morbidity. *Addict Biol*. (2015) 20:756-72. doi: 10.1111/adb.12156

23. Muylaert DE, van Almen GC, Talacua H, Fledderus JO, Kluin J, Hendrikse SI, et al. Early in-situ cellularization of a supramolecular vascular graft is modified by synthetic stromal cell-derived factor-1α derived peptides. *Biomaterials*. (2016) 76:187-95. doi: 10.1016/j.biomaterials.2015.10.052

24. Boyarchuk O, Romanyshyn Y, Savchak I, Kravets V, Shymanska I, Makukh H. Two case reports of B-cell lymphopenia associated with IGLL1 variants identified through newborn screening in Ukraine. *Front Pediatr*. (2025) 13:1566867. doi: 10.3389/fped.2025.1566867

25. Baxter RC. Endocrine and cellular physiology and pathology of the insulin-like growth factor acid-labile subunit. *Nat Rev Endocrinol*. (2024) 20:414-25. doi: 10.1038/s41574-024-00970-4

26. Esteghamat F, Broughton JS, Smith E, Cardone R, Tyagi T, Guerra M, et al. CELA2A mutations predispose to early-onset atherosclerosis and metabolic syndrome and affect plasma insulin and platelet activation. *Nat Genet*. (2019) 51:1233-43. doi: 10.1038/s41588-019-0470-3

27. Motta JP, Rolland C, Edir A, Florence AC, Sagnat D, Bonnart C, et al. Epithelial production of elastase is increased in inflammatory bowel disease and causes mucosal inflammation. *Mucosal Immunol*. (2021) 14:667-78. doi: 10.1038/s41385-021-00375-w

28. Mauri N, Kleiter M, Dietschi E, Leschnik M, Högler S, Wiedmer M, et al. A SINE insertion in ATP1B2 in Belgian shepherd dogs affected by spongy degeneration with cerebellar ataxia (SDCA2). *G3 (Bethesda)*. (2017) 7:2729-37. doi: 10.1534/g3.117.043018

29. Boer K, Spliet WG, van Rijen PC, Jansen FE, Aronica E. Expression patterns of AMOG in developing human cortex and malformations of cortical development. *Epilepsy Res*. (2010) 91:84-93. doi: 10.1016/j.eplepsyres.2010.06.015

30. Kamizaki K, Doi R, Hayashi M, Saji T, Kanagawa M, Toda T, et al. The Ror1 receptor tyrosine kinase plays a critical role in regulating satellite cell proliferation during regeneration of injured muscle. *J Biol Chem*. (2017) 292:15939-51. doi: 10.1074/jbc.M117.785709
